# Clinical and epidemiological correlates of antibody response to human papillomaviruses (HPVs) as measured by a novel ELISA based on denatured recombinant HPV16 late (L) and early (E) antigens

**DOI:** 10.1186/1750-9378-3-9

**Published:** 2008-06-26

**Authors:** Colomba Giorgi, Paola Di Bonito, Felicia Grasso, Stefania Mochi, Luisa Accardi, Maria Gabriella Donà, Margherita Branca, Silvano Costa, Luciano Mariani, Alberto Agarossi, Marco Ciotti, Kari Syrjänen, the HPV-PathogenISS group.

**Affiliations:** 1 Department of Infectious, Parasitic and Immunomediated Diseases, Istituto Superiore di Sanità, Roma, Italy; 2 Unità Citoistopatologia, Centro Nazionale di Epidemiologia, Sorveglianza e Promozione della Salute, Istituto Superiore di Sanità, Roma, Italy; 3 Dipartimento di Ginecologia e Ostetricia, Ospedale S. Orsola Malpighi, Bologna, Italy; 4 Clinica Ostetrica e Ginecologica, IFO, Istituto Regina Elena, Roma, Italy; 5 Istituto Scienze Biomediche, Ospedale Luigi Sacco, Milano, Italy; 6 Laboratory of Clinical Microbiology and Virology, Policlinico of Tor Vergata University, Rome, Italy; 7 Department of Oncology and Radiotherapy, Turku University Hospital, FIN-20521 Turku, Finland

## Abstract

**Background:**

At present, seroreactivity is not a valuable parameter for diagnosis of Human Papillomavirus (HPV) infection but, it is potentially valuable as marker of viral exposure in elucidating the natural history of this infection. More data are needed to asses the clinical relevance of serological response to HPV.

**Objectives:**

The objective was to assess the clinical and epidemiological correlates of HPV-seroreactivity in a cohort of HIV-negative and HIV-positive women.

**Methods:**

Seroreactivity of 96 women, evaluated in an ELISA test based on denatured HPV16 late (L) and early (E) antigens, was correlated with their clinical and epidemiological data previously collected for a multi-centre Italian study, HPV-PathogenISS study.

**Results:**

No significant correlation was found between HPV DNA detection and seroreactivity. Women, current smokers showed significantly less seroreactivity to L antigens as compared with the non-smokers. HIV-positive women showed significantly less (66.7%) antibody response as compared with HIV-negative women (89.3%), with particularly impaired response to L antigens. Women, HIV-positive and current smokers, showed by far the lowest seroprevalence (33.3%) as compared to 75.9% among all other women (OR = 0.158; 95%CI 0.036–0.695, p = 0.014; Fisher's exact test). Importantly, this association did not loose its significance when controlled for confounding from age (continuous variable) in multivariate analysis or using Mantel-Haenszel test for age-groups.

**Conclusion:**

It is tempting to speculate that HIV-positive current smokers comprise a special high-risk group, with highly impaired immunological response that could prevent eradication of persistent HPV infections and thus contribute to development of CIN3/CC.

## Background

Human Papillomavirus (HPV) infection, known to be associated with both benign and malignant tumours at different mucosal sites, is widespread among female and male populations [1,2]. The oncogenic high-risk HPV types (HR-HPVs) are the single most important etiological agents of cervical cancer (CC) and its precursor (CIN) lesions [3,4]. Despite global differences in the prevalence of individual HR-HPV types, HPV16 remains the most frequent HR-HPV type in all geographic regions and the current HPV research is mostly focused on it [3,4]. Diagnosis of HPV infection is based on detection of viral DNA using different PCR-based methods or commercially available hybrid capture (HC2) test [5,6]. Until now, serological studies have had little to offer in diagnosis of HPV infections [7]. The first serological assays for HPV antibody detection were introduced in the early 1970's [8], but the more widespread use of HPV serology had to await the development of more refined assays based on virus-like particles (VLP) in the early 1990's [9,10]. A growing body of serology data has being obtained using different viral protein-based ELISA to analyse HPV antibodies in different settings, most notably in large-scale seroepidemilogical- and case-control studies to assess the risk factors of cervical carcinoma (CC) in HPV exposed subjects [11-17]. The main conclusion from the plethora of sero-epidemiological studies is that antibody response to viral proteins does not invariably occur during a natural HPV infection [14,15]; only half of HPV DNA-positive women with normal cytology shows antibody response to VLPs [18,19]. HPV E6/E7 antibodies are predominantly found in patients with CC, but they are also detected in healthy controls, precluding the use of their detection as predictor of CC [14,15,20].

VLP-based assays have been recognized as reliable genotype-specific assays [14,15,21] and potentially will contribute to the understanding of the natural history of HPV infections [18,19,22]. New L1VLP-based vaccines have been shown to induce levels of HPV type specific antibodies higher than that found in natural infected women [23]. Correlation between anti-L1VLPs antibodies and protection from reinfection in natural infection is still a controversial issue [24,25]. An important problem with serological studies resides in the fact that the results from different laboratories are not comparable for the lack of reference specific sera [26]. For the serological response evaluation of the new HPV vaccines the WHO recommends the standardisation of the different in-house L1-based serological methods by the use of reference sera and promoted an international collaborative study with this proposal [21].

To improve the understanding of correlation between infection and antibody response in patients with and without cervical lesions, prospective cohort studies using a widespread panel of viral antigens would be needed [14,15,26]. Similarly, it would be necessary to study the dynamics of HPV-E and -L antigen-specific antibodies in relation to their clinical and epidemiological correlates [27,28], including immune-suppression due to HIV infection [25,29]. There are recent growing evidences regarding the induction of antibodies neutralising across papillomavirus types after vaccination with VLP displaying L2 epitopes or with fusion protein containing L2E7E6 viral proteins or synthetic peptides of the HPV16 L2 protein [30-32]. Very recently Rizk and collaborators [33] have shown that L1 fused to GST protein displays a broad variety of epitopes, both conformational and linear; the neutralising ones are conformational and, mostly, type specific while the cross-reacting ones are associated with linear epitopes.

We have recently reported the development of an in-house ELISA test based on five recombinant HPV16 proteins expressed in E. coli: L1 and L2 capsid proteins, E6 and E7 oncoproteins and the non-structural E4 protein, all used in unfolded form [34]. The idea of using denatured antigens comes from the observation of a high amino acid identity, ranging from 36% for E6 to 74% for L1, among the same proteins belonging to different HPV genotypes and then the possibility to detect cross-reacting antibodies directed against linear epitopes. By this assay we were able to detect an antibody response in women infected with HPV genotypes different from that of the reference antigen. In this study we also determined the Avidity Index (AI) [35] of the positive samples. Antibody response to linear epitopes has been reported in the past [36,37] and was correlated to the specific stage of HPV-associated cervical disease [38]; nevertheless this antibody response can not be related to protection level against HPV infection, that is associated to the neutralising component of humoral response. Our system, revealing antibodies only against linear epitopes, could be utilized to evaluate the immunological responsiveness of subjects to HPV infection and contribute to estimate the risk of infection progression but it could not provide information about protection level from reinfection in both natural infection and after vaccination.

In the present study we have correlated the antibody response, evaluated with our system, to the baseline epidemiological, clinical and HPV DNA data of women enrolled in a multi-centre cohort study designed to explore pathogenetic mechanisms and prognostic factors of CIN lesions in HIV-positive and HIV-negative women, known as HPV-PathogenISS study [39].

## Results

### Correlation of epidemiological characteristics to serological response

The recorded epidemiological characteristics of the enrolled women have been correlated to their serological response to either all HPV16 antigens, or separately E and L proteins (Table 1). The statistical analysis shows that antibody response is not related to patient's age or their ethnic origin (data not shown); however, the power of the study to detect true differences in the age between the two groups is limited, due to the small sample size. Interestingly, current smokers have significantly (p = 0.009) less seroreactivity to L antigens as compared with the non-smokers (OR = 0.26; 95%CI 0.095–0.764), whereas the difference in reactivity to E and E+L antigens is not significant. No differences in seroreactivity could be attributed to the other smoking variables (age at onset, number of cigarettes/day, smoking years) (data not shown), or to any of the indicators of obstetric history or sexual behaviour, including contraception used in the past. Women's seroreactivity was not influenced by their being sexually active, their having a new partner since the last visit in the clinic, or their having a HIV-positive sex partner. Interestingly, the actual mode of contraception itself was not significant, but the different practice of condom use was significantly (p = 0.022) related to seroreactivity to L antigens, being the least frequent among women using always condom in sexual intercourse.

**Table 1:** Key epidemiological data of patients related to their serological response to HPV16 antigens

**Variables**	**Anti-E and -L**			**Anti-E_4_-E_6 _and E_7_**			**Anti L_1 _and -L_2_**		
			
	**+**	**-**	**Sign.**	**+**	**-**	**Sign.**	**+**	**-**	**Sign.**
**Mean Age (yrs)**	34.9	36.9	p = 0.460	34.8	36.0	p = 0.533	36.1	35.6	p = 0.805
**Smoking status**	%								
Non-smoker (40)	92.5	7.5	OR = 2.8 (0.7–11.3);	72.5	17.5	OR = 1.4 (0.6–3.6);	85.0	15.0	OR = 3.7 (1.3–10.5);
			p = 0.211			p = 0.495			**p = 0.009**
Current smoker (48)	81.3	18.7		64.6	35.4		60.4	39.6	
**N. cigarette/d**	13.4	10.1	p = 0.285	13.8	10.9	p = 0.225	13.3	12.0	p = 0.603
**Age start smoking (yrs)**	18.9	19.6	p = 0.776	18.2	20.6	p = 0.122	19.5	18.1	p = 0.379
**Years smoked**	15.2	13.3	p = 0.592	16.5	12.0	p = 0.089	14.0	16.8	p = 0.296
**^1^Contraception (%):**									
Yes (53)	92.5	7.5	OR = 1.4 (0.3–6.8);	73.6	26.4	OR = 1.3 (0.5–3.4):	73.6	26.4	OR = 0.7 (0.2–2.1):
			p = 0.694			p = 0.658			p = 0.560
No (29)	89.7	10.3		69.0	31.0		79.3	20.7	
**^New partner (%):**									
Yes (5)	100	-	OR = NC; p = 0.508	60.0	40.0	OR = 0.7 (0.2–2.2);	60.0	40.0	OR = 0.7 (0.2–2.1);
						p = 0.630			p = 0.619
No (76)	86.8	13.2		71.1	28.9		72.4	27.6	
**Sexually active (%):**									
Yes (79)	89.9	10.1	OR = 5.9 (0.9–40.9);	70.9	29.1	OR = 1.6 (0.3–10.4);	74.7	25.3	OR = 4.4 (0.7–28.4);
			p = 0.105			p = 0.631			p = 0.124
No (5)	60.0	40.0		60.0	40.0		40.0	60.0	
**Partner HIV+ (%):**									
Yes (8)	87.5	12.5	OR = 0.9 (0.1–8.7);	87.5	12.5	OR = 3.3 (0.4–28.3);	62.5	37.5	OR = 0.6 (0.1–2.8);
			p = 1.000						p = 0.679
No (75)	88.0	12.0		68.0	32.0		73.3	26.7	
**^2^Actual contraception**	%								
Yes (37)	86.5	13.5	OR = 0.7 (0.2–2.7);	67.6	32.4	OR = 0.8 (0.3–2.1);	64.9	35.1	OR = 0.4 (0.1–1.1);
			p = 0.726			p = 0.660			**p = 0.093**
No (43)	90.7	9.3		72.1	27.9		81.4	18.6	
**^2^Condom Use**	%								
Never (43)	90.7	9.3		72.1	27.9		72.1	27.9	
Often (14)	100	-	p = 0.138	78.6	21.4	p = 0.334	100	-	**p = 0.022**
Always (18)	77.8	22.2		55.6	44.4		61.1	38.9	
**STD since last visit**	%								
Yes (6)	100	-	OR = NC;	100	-	OR = NC;	83.3	16.7	OR = 1.7 (0.2–15.7)
			p = 1.000			p = 0.175			
No (74)	89.2	10.8		68.9	31.1		74.3	25.7	

NC, non-computable; ^1^Contraception used until past; ^2^Contraception in current use; *For linear trend; HPV-NCIN, HPV infection without CIN.... ^New partner since last visit

### Correlation of clinical data to serological response

The key clinical data were then related to HPV seroreactivity; the results are shown in Table 2. Seroreactivity to E, L or E & L antigens seems to be not associated with HPV DNA positivity, either for all HPVs or for LR-HPV and HR-HPV genotypes separately. Other baseline clinical data, as the reported past HPV infection, abnormality in the referral Pap smear and grade of CIN, were analysed regarding the sero-status. Only a reported past HPV infection was associated with significantly (p = 0.046) higher probability of having antibodies to E antigens (OR = 4.8; 95%CI 1.1–22.8). No statistical correlation was found between the sero-status and the other considered clinical data, as the presence of external genital warts (Condylomata acuminata) or their number (data not shown). Mild abnormality on colposcopy was found associated with significantly increased seropositivity to L antigens, as compared with normal or high-grade colposcopy (p = 0.015), no such difference was found in reactivity to E or to E+ L antigens.

**Table 2:** Key clinical characteristics of the patients related to their serological response to HPV16 antigens

**Variables**	**Anti-E and -L**			**Anti-E_4_-E_6 _and E_7_**			**Anti L_1 _and -L_2_**		
			
	**+**	**-**	**Sign.**	**+**	**-**	**Sign.**	**+**	**-**	**Sign.**
**HPV DNA (%):**									
Positive 76	89.5	10.5	OR = 2.8 (0.8–9.9)	71.1	28.9	OR = 1.3 (0.4–3.7)	71.1	28.9	OR = 1.6 (0.6–4.6)
			p = 0.136			p = 0.604			p = 0.350
Negative 20	75.0	25.0		65.0	35.0		60.0	40.0	
**HR-HPV (%)**									
Positive 66	87.9	12.1	OR = 1.4 (0.4–4.8)	68.2	31.8	OR = 0.8 (0.3–2.0)	69.7	30.3	OR = 1.1 (0.5–2.9)
			p = 0.536			p = 0.608			p = 0.767
Negative 30	83.3	16.7		73.3	26.7		66.7	33.3	
**LR-HPV (%):**									
Positive 10	100	0.0	OR = NC	90.0	10.0	OR = 4.3 (0.5–30.0)	80.0	20.0	OR = 1.9 (0.4–9.7):
			p = 0.348			p = 0.273			p = 0.720
Negative 86	84.9	15.1		67.4	32.6		67.4	32.6	
**Previous:**									
**HPV (%):**									
Yes 19	94.7	5.3	OR = 2.8 (0.3–23.9);	89.5	10.5	OR = 4.8 (1.1–22.8)	73.7	26.3	OR = 0.9 (0.3–3.1);
			p = 0.445			**p = 0.046**			p = 0.961
No 66	86.4	13.6		63.6	36.4		74.2	25.8	
**HIV-Status (%):**									
Negative 84	89.3	10.7	OR = 4.2(1.1–16.5)	71.4	28.6	OR = 1.8 (0.5–6.2)	72.6	27.4	OR = 3.7 (1.1–12.9)
			**p = 0.032**			p = 0.355			**p = 0.030**
Positive 12	66.7	33.3		58.3	41.7		41.7	58.3	
Smokers 9	66.6	33.3		55.6	44.4		33.3	66.7	
**Previous:**									
**PAP (%):**									
Never 10	90.0	10.0	p = 0.793	70.0	30.0	p = 0.111	80.0	20.0	p = 0.445
Irregular 26	92.3	7.7		84.6	15.4		61.5	38.5	
Regular (1–3 yrs) 49	85.7	14.3		61.2	38.8		75.5	24.5	
**Referral**									
**PAP (%):**									
ASC-US 7	100	0.0	p = 0.431	85.7	14.3	p = 0.623	100	0.0	p = 0.094
LSIL 48	91.7	8.3		64.6	35.4		75.0	25.0	p = 0.031*
HSIL 16	81.3	18.7		68.8	31.2		56.3	43.7	
**Biopsy**									
**Result (%):**									
Normal 1	100	0.0		0.0	100		100	0.0	
HPV-NCIN 9	100	0.0	p = 0.262	66.7	33.3	p = 0.526	77.8	22.2	p = 0.564
CIN1 11	90.9	9.1		54.5	45.5		63.6	36.4	
CIN2 14	92.9	7.1		71.4	28.6		85.7	14.3	
CIN3 15	93.3	6.7		73.3	16.7		73.3	26.7	
SCC 2	50.0	50.0		50.0	50.0		50.0	50.0	
**Genital**									
**Warts (%):**									
Yes 20	95.0	5.0	OR = 3.3(0.4–27.7)	80.0	20.0	OR = 2.1 (0.6–7.0)	75.0	25.0	OR = 1.1 (0.4–3.7)
			p = 0.437			p = 0.275			p = 0.801
No 61	85.2	14.8		65.6	34.4		72.1	27.9	
**Colposcopy (%):**									
Normal 2	100	0.0		100	0.0		0.0	100	
Low-grade 27	96.3	3.74	p = 0.177	63.0	37.0	p = 0.668	85.2	14.8	**p = 0.015**
High-grade 28	77.8	22.2		72.2	27.8		61.1	28.9	

NC, non-computable; ^1^Contraception used until past; ^2^Contraception in current use; *For linear trend; HPV-NCIN, HPV infection without CIN....

The key epidemiological and clinical data were also related to the AI values of L and E antigens determined in a previous study [34]. This is a useful parameter used in a large number of viral diseases to discriminate between low and high antibody avidity; 40% is considered the discrimination AI value [40-42]. In our system AI has been considered as an indirect measure of the specific interaction between antigen and cross-reacting antibodies. Most reactions with L1, L2, E4 and E7 antigens showed an AI higher than 40%, but not those reacting with E6 antigen. Few significant associations were observed between high AI and the different epidemiological and clinical variables. High AI of E7 antibodies was associated with 100% of women who did not use contraception in the past, as compared with 80% among women who used regular contraception (p = 0.044). Similarly, regular use of condom was marginally (p = 0.076) associated with lower probability of high-avidity antibodies to L1. None of HR-HPV negative women had high-avidity antibodies to E6, as compared with 29.4% of HR-HPV positive women (p = 0.059). Interestingly, women with normal colposcopy had the highest AI for E7, followed by those with high-grade colposcopy (77.1%) and low-grade abnormality (54.1%) (p = 0.011). AI was not related to HIV/smoking status, being practically identical between the two groups for both L and E antigens.

### Correlation of HIV status to serological response

When the HIV status was taken into account (Table 2), HIV-positive women showed less seroreactivity to E+L or E and L separately than the HIV-negative ones. Interestingly only the difference of seroreactivity to L antigens reached statistical significance (Fisher's exact test; p = 0.030).

HIV-positive and current smoker women, showed the lowest L seroprevalence (33.3%), as compared with 75.9% among all other women (p = 0.014; Fisher's exact test), with OR = 0.158 (95%CI 0.036–0.695). This association was not confounded by age, when tested either using Mantel-Haenszel test for age groups (common OR = 0.178; 95%CI 0.038–0.832) (p = 0.028) or logistic regression analysis for age as continuous variable (adjusted OR = 0.167; 95%CI 0.037–0.736) (p = 0.020). On the other way round, women who are HIV-negative and not smokers are significantly more likely to mount antibody response (OR = 6.31; 95%CI 1.43–27.71).

## Discussion

In a previous study we have reported the baseline data of a cohort comprising HIV-positive and HIV-negative women [39]. The present report provides data on HPV exposure status of a group of these women that gave their blood. The analysis regards the serological response to recombinant HPV16 L1, L2, E4, E6 and E7 proteins evaluated by a new ELISA technique [34].

We have correlated the women's epidemiological characteristics to their serological response to HPV16 antigens, using three possible outcomes; i) seroreactivity to either E or L (= HPV seropositive or not), ii) reactivity to E, and iii) reactivity to L proteins. Only a few of the recorded epidemiological variables proved to be significantly related to serostatus of the women in this cohort. Interestingly current smokers showed significantly less seroreactivity to L antigens as compared with the non-smokers (OR = 3.7; 95%CI 1.3–10.5) (p = 0.009), while the difference in reactivity to E and E+L antigens was not significant. This is in full agreement with a recent report, where almost identical observations were reported [28]. In this study, smokers were twice as likely as non-smokers to test negative for anti-HPV L1 antibodies, even when the effects of other covariates were considered in the analysis (adjusted OR = 0.5; 95%CI 0.2–0.9). The relationship between tobacco smoking and CC is highly complex; however, the basic mechanisms must involve the host's inability to clear HR-HPV infection. The observed failure of smokers to mount antibody response to L antigens could be an indication of the deregulation of immune responses induced by smoking, increasing the likelihood of persistent infection and the long-term risk for CC. This is in agreement with other results showing current smoking to be associated with persistent HR-HPV infection [43,44].

The relationship of contraception and HPV infection has been incompletely studied and only in relation with HPV DNA detection [27,45,46], but not in relation to antibody detection. In the present analysis only the condom use, among the recorded epidemiological variables, was related to seroreactivity to L antigens; the lowest (61.1%) seropositivity (Table 1) was observed in those women who reported to always use condom in sexual intercourse.

The relationship between HPV DNA detection and antibody response to different HPV antigens is highly complex. Large sero-epidemiological studies seem indicate that antibody response to viral antigens does not invariably occur during natural HPV infection [14,15]. Similarly, only approximately half of HPV DNA-positive women with normal cytology show antibody response to VLPs [18,19]. Moreover E6/E7 antibodies are detected in patients with CC as well as in healthy controls. [14,15,47]. These observations are confirmed in the present cohort, where detection of HPV DNA, both LR+HR genotypes and LR- and HR-HPV separately, did not bear any significant relationship with seroreactivity to any viral antigens. However women who reported past HPV infection show a significantly higher prevalence (89.5%) of E antibodies as compared with that (63.6%) of women who did not reported past infection (OR = 4.8; 95%CI 1.1–22.8) (Table 2). These results suggest that seropositivity to E antigens could be considered as a surrogate for past viral infection [48].

Into the HPV-PathogenISS cohort, HIV-positive women were also enrolled [39]. As generally accepted, these women are at increased risk for progression to CIN3 and CC [49], mainly because of their failure to eradicate persistent HR-HPV infection, even if treated by HAART [50]. HPV serology in HIV-positive women has been studied only recently. In these women we have observed a significantly lower seroprevalance compared to the HIV-negative ones; these data are in contrast with those reported in large cohort studies, where HIV-positive women usually have higher prevalence of HPV antibodies [51,25,29]. Some reasons of this discrepancy can be the small number of HIV-positive women in our cohort leaving room for observations by chance and the fact that all patients had been on HAART for several years and their HPV baseline status was very similar to that of HIV-negative women. On the other hand, this observation could indicate their lower HPV exposure in the past. A feasible explanation could be the HIV-induced immunosuppression leading to perturbed early immune responses against HPV [50]; unfortunately, the CD4 data of these women are incomplete and it is not possible to draw firm conclusions on their level of immunosuppression. With regard to the above discussed failure of smokers to mount viral antibody response, we analysed the combined effect of current smoking and HIV-status in this cohort. Based on the obtained results and our previously reported data [50,44], it is tempting to speculate that HIV-positive women who are current smokers comprise a special high-risk group, with extremely high probability of developing CIN3/CC, due to their failure to eradicate persistent HR-HPV infection. This failure could be attributed to their inability to mount effective immunological responses against HPV antigens, of which deregulation the low seroreactivity is an indication.

Two other observations merit some discussion. We noticed that seroresponse to L proteins declined in parallel with the increasing severity of abnormality in the referral PAP smear, from 100% among ASCUS cases to 56.3% among those with HSIL (Table 2)(p = 0.031 for linear trend). This could substantiate the observations of Wiley and collabotators [28] who reported that women with ASCUS were twice more likely to test anti-HPV L1-positive as compared with those with normal PAP. In our cohort, we had no PAP-negative women to confirm this.

As recently discussed [34], most of the HPV antibodies bound to these 5 antigens with an AI higher than 40%, even in the sera from women infected with HPV type different from 16 type. When related to epidemiological data and clinical observations, AI data yielded few significant observations. No feasible explanation can be offered for the association of high AI of anti-E7 antibodies in 100% of women which did not use contraception in the past, as compared with the 80% of those using contraception (p = 0.044). Similarly, the significant difference (p = 0.011) obtained in AI between the different colposcopy categories does not show any linear trend with the grade of abnormality.

## Conclusion

In summary the results show that there is no meaningful relationship between HPV DNA detection and seroreactivity to tested HPV antigens, which was not unexpected in the light of published data [14,15,19]. However, two significant observations are of particular interest. Current smokers show significantly (p = 0.009) less seroreactivity to HPV L antigens as compared with the non-smokers. The difficulty of smokers to mount antibody response to L antigens could be an indication of a marked deregulation of the immune system responses thus increasing the long-term risk for CIN3/CC. Another interesting observation is the significantly impaired seroreactivity to HPV L antigens among HIV positive women, further accentuated among women HIV positive and current smokers. It is tempting to speculate that HIV positive women who are current smokers comprise a special high-risk group, with extremely high probability of developing CIN3/CC, due to their failure to eradicate persistent HR-HPV infection.

## Methods

### Patients

In the HPV-PathogenISS study a cohort of 286 women were enrolled during 2002–2003 by Gynaecologic outpatient departments at 5 hospitals (S. Orsola Malpighi, Bologna; Istituto Regina Elena, Roma; Università di Tor Vergata, Roma; Luigi Sacco, Milano; and Istituto San Gallicano, Roma). Women were selected for having abnormal PAP test (ASC-US, LSIL or HSIL) [39]. The sera examined in this study are from a group of 96 women of this cohort that gave the informed consent for serum taking for research use. All women were subjected to colposcopy, PAP test (if not taken within the past 3 months), biopsy (when indicated), as well as sampling for HPV DNA testing. This study protocol was separately approved by the institutional Ethical Committees of all 5 hospitals. Epidemiological interview, Pap test, colposcopy, biopsy, treatment by LLETZ are been previously described [39].

### Sampling for HPV testing and HPV genotype identification

A careful sampling protocol for HPV testing was followed in all clinics. Both exo- and endocervical specimens were collected and stored at -70°C, until transported to the two virology laboratories (Tor Vergata and ISS) for HPV analysis. HPV genotyping was performed by sequence analysis, as previously described [34]. When the genotype was not resolved by sequencing the samples were examined for the presence of either HR- or LR- HPV by HC2 assay (Digene). Because the genotype determination was not possible for all samples the analysis reported in this paper have been done by considering only the HR- and LR-HPV categories.

### HIV testing

The patient HIV-serostatus was determined at the entry with ELISA test and confirmed by Western blot, with the informed consent of the patients. The state of HIV disease in sero-positive patients was assessed by CD4+ and CD8+ lymphocytes counting and recording the opportunistic infections or neoplasia. Unfortunately CD4 parameter was not available for all HIV-positive patients. All these patients were being treated with highly active anti-retroviral treatment (HAART). HIV-positive patients in this study were only 12, of whom 9 current smokers.

#### HPV serology

##### Human sera and control sera

The collection and treatment of sera of the 96 women have been previously described [34]. Control sera were randomly taken among sera collected for studies unrelated with HPV infection. As positive controls, antigen specific hyper-immune sera obtained by injection in either mice or rabbits of the recombinant purified HPV16 L1, L2, E4, E6 and E7 proteins were used [34].

##### ELISA and avidity test

The presence in the sera of anti-HPV antibodies was evaluated by an ELISA test based on the recombinant L1, L2, E4, E6 and E7 HPV16 proteins [34]. A cut-off value for each antigen was calculated as the arithmetic mean of the absorbance values of 40 control sera, plus two standard deviations (SD). After a first calculation the outlier values were excluded and the cut-off recalculated [52]. The reproducibility of ELISA results was assayed by testing each serum in duplicate and repeating each test three times.

Avidity of the specific anti-HPV IgGs was evaluated and the AI, expressed as a percentage of the absorbance in ELISA with urea wash and the absorbance in ELISA without urea wash × 100 [35]. Because of the lack of data regarding the avidity antibodies in HPV infection, it was difficult to establish an AI value discriminating between low and high avidity. We tentatively assumed the 40% value, a discriminating value reported for some infectious diseases [40-42].

### Statistical analyses

Statistical analyses were performed using the SPSS^® ^software package (SPSS for Windows, version 14.01). Frequency tables were analysed using the Chi-square test, with likelihood ratio (LR) or Fisher's exact test being used to assess the correlation between categorical variables. OR and 95% confidence intervals (95%CI) were calculated where appropriate, using the exact method. Differences in the means of continuous variables were analysed using non-parametric tests (Mann-Whitney or Kruskal-Wallis) or ANOVA (to obtain the mean values). Logistic regression analysis was used to control for the confounding from age to some observed significant univariate predictors of seroreactivity, e.g. HIV/smoking status. Confounding was also controlled by calculating the common odds ratio (OR) for the stratum-specific estimates, using the Mantel-Haenszel test. To evaluate the statistical power of the study (e.g. for age differences), we used STATA/SE 9.2. power analysis option, with the default value (0.05) for alpha, and using the true sample sizes of the women in the two groups, e.g. seropositive and -negative women. In all tests, the values p < 0.05 were regarded statistically significant.

## Authors' contributions

CG conceived the study, participated in his design and coordination and in rafting the manuscript, PDB, FG, SM, MGD participated in acquisition analysis and interpretation of serology data, LA participated in interpretation of data and critically revised the manuscript, MB, SC, LM, AA, MC participated in acquisition of serum samples and epidemiological data, KS participated in design of the study, performed the statistical analysis and participated in drafting the manuscript.
